# Microendoscopy in vivo for the pathological diagnosis of cervical precancerous lesions and early cervical cancer

**DOI:** 10.1186/s13027-023-00498-8

**Published:** 2023-04-26

**Authors:** Min Liu, Jianqiao Lu, Yong Zhi, Yetian Ruan, Guangxu Cao, Xinxin Xu, Xin An, Jinli Gao, Fang Li

**Affiliations:** 1 Department of Obstetrics and Gynecology, Shanghai East Hospital, School of Medicine, Tongji University, 150 Jimo Road, Shanghai, 200120 China; 2 OptoMedic Technologies Inc., Foshan, 528200 China; 3 Department of Pathology, Shanghai East Hospital, School of Medicine, Tongji University, Shanghai, 200120 China

**Keywords:** Microendoscopy, Cervical intraepithelial neoplasia, Cervical cancer, Colposcopy, Optical biopsy, Cervical biopsy

## Abstract

**Background:**

Cervical cancer is an important public health problem. Conventional colposcopy is inefficient in the diagnosis of cervical lesions and massive biopsies result in trauma. There is an urgent need for a new clinical strategy to triage women with abnormal cervical screening results immediately and effectively. In this study, the high-resolution microendoscopy combined with methylene blue cell staining technology was used to perform real-time in vivo imaging of the cervix for the first time.

**Methods:**

A total of 41 patients were enrolled in the study. All patients underwent routine colposcopy and cervical biopsy, and high-resolution images of methylene blue-stained cervical lesions were obtained in vivo using microendoscopy. The cell morphological features of benign and neoplastic cervical lesions stained with methylene blue under microendoscopy were analyzed and summarized. The microendoscopy and histopathology findings of the high-grade squamous intraepithelial lesion (HSIL) and more severe lesions were compared.

**Results:**

The overall consistency of microendoscopy diagnosis with pathological diagnosis was 95.12% (39/41). Diagnostic cell morphological features of cervicitis, low-grade squamous intraepithelial lesion (LSIL), HSIL, adenocarcinoma in situ, and invasive cancer were clearly demonstrated in methylene blue stained microendoscopic images. In HSIL and more severe lesions, microendoscopic methylene blue cell staining technology can show the microscopic diagnostic features consistent with histopathology.

**Conclusions:**

This study was an initial exercise in the application of the microendoscopy imaging system combined with methylene blue cell staining technology to cervical precancerous lesions and cervical cancer screening. The results provided the basis for a novel clinical strategy for triage of women with abnormal cervical screening results using in vivo non-invasive optical diagnosis technology.

**Supplementary Information:**

The online version contains supplementary material available at 10.1186/s13027-023-00498-8.

## Introduction

Cervical cancer is the fourth leading cause of cancer deaths in women, with the majority of cases occurring in developing countries and regions [1]. In China, with 109,741 new cases and 59,060 deaths in 2020, cervical cancer has become an important public health problem [1]. Cervical precancerous lesions and early-stage cervical cancer can be cured by surgery, while the prognosis of advanced cervical cancer is poor. The 5-year progression-free survival rates of stage II, III, and IV cervical cancer are only 71%, 55%, and 16%, respectively [2, 3]. Therefore, strengthening the accurate identification of cervical intraepithelial neoplasia (CIN)/early-stage cervical cancer has critical clinical significance for eliminating cervical cancer.

Currently, cytology and human papillomavirus (HPV) testing are the main screening methods for cervical cancer. Women with abnormal screening results need to undergo colposcopy and biopsy. Pathological biopsy under colposcopy is the gold standard for CIN/cervical cancer diagnosis, but this diagnostic method has limitations in clinical application. The conventional colposcopy diagnosis is subjective, inaccurate, and has a high rate of missed diagnosis [4–6]. However, a tissue biopsy has trauma to the cervix, which can lead to complications such as bleeding and infection. Moreover, the return time of biopsy pathological reports is long, resulting in a large number of patients being lost to follow-up [7, 8]. In addition, it is worth noting that high-risk HPV infection in the cervix is very common and most infections can be cleared by the autoimmune system within 1–2 years. Only long-term persistent high-risk HPV infection can cause CIN2 and more severe lesions, which, coupled with the high sensitivity of HPV testing, leads to the significant phenomenon of excessive biopsy in clinical practice [9–12]. In view of the above limitations of colposcopy and histopathological examinations, there is an urgent need for an immediately effective clinical strategy for the triage of women with abnormal cervical screening results.

The development of optical imaging technology allows us to assess human diseases at the microscopic level in real time [13]. High-resolution microendoscopy, as a representative new imaging method, can perform real-time, in vivo imaging of epithelial tissues at the microvascular and subcellular levels, and can be used in conjunction with standard endoscopy to reduce costs. This new tool for non-invasive immediate “optical biopsy” of precancerous lesions/cancers has shown good prospects in the screening and triage of cervical cancer, head and neck cancer, and gastrointestinal cancer [14–16]. However, there are still some problems with these microendoscopy techniques, including limited imaging penetration depth, lack of deep tissue resolution, insufficient visual field, etc., which require further research and improvement [7, 14]. We have developed a high-resolution microendoscopy imaging system that allows real-time in vivo diagnosis of cervical epithelium based on pathological vascular features without staining, and previous clinical study has shown its great value for the diagnosis of cervical lesions [17].

In pathology, nuclear morphological changes are used as the diagnostic “gold standard” to distinguish benign and malignant cells. Nuclear morphological changes are mainly manifested as increased nuclear size, increased nuclear-to-cytoplasmic ratio, irregular nuclear membrane, and abnormal chromatin distribution et al. [18, 19]. Changes in nuclear morphology are widely related to the dynamics and function of cancer cells, and cervical precancerous lesions and carcinoma frequently lead to specific changes in nuclear morphology [19]. Therefore, the combination of optical imaging technology and nuclear morphology detection is a new direction to achieve non-invasive in vivo real-time “optical diagnosis” of cervical lesions by microendoscopy.

In this study, we combined the microendoscopic imaging system with methylene blue cell staining technique to perform in vivo cervical imaging of patients. For the first time, we reported microendoscopy images of methylene blue-stained cervical lesions at cellular resolution, analyzed the cell morphological features for cervical precancerous lesions and cervical cancer, and further compared these features with histopathology. These results verified the feasibility of the microendoscopic methylene blue cell staining technique in the diagnosis of cervical precancerous lesions/cervical cancer and established the diagnostic criteria for microendoscopy images of CIN2 and more serious lesions. This study will lay the foundation for future clinical applications of non-invasive optical diagnosis in the future.

## Materials and methods

### Eligible patients

The study has gained the approval of the Institutional Ethics Committee of Shanghai East Hospital, School of Medicine, Tongji University (No:2021 − 194). A total of 41 women with abnormal cervical screening results who visited the Department of Obstetrics and Gynecology, Shanghai East Hospital, School of Medicine, Tongji University, were included in this study from June 2021 to December 2021. All subjects were older than 18 years old, voluntarily underwent microendoscopy, and signed written informed consent. According to the 2017 American Society for Colposcopy and Cervical Pathology (ASCCP) colposcopy standard and clinical research requirements, the following inclusion criteria were set: positive for HPV16/18, persistent positive results for other types of high-risk HPV for more than one-year, abnormal cytology results. Exclusion criteria: known pregnancy, HIV infection or AIDS, previous hysterectomy, receiving (or having previously received) radiation therapy or chemotherapy for cervical cancer (or other cancer), severe heart, lung, liver, kidney dysfunction, and failure to follow up on time.

### Microendoscopy

The microendoscopy system (Opto-Medic, Guangdong, China) used in this study is an optical microscope that enables real-time cellular-resolution imaging of cervical epithelial tissue in vivo. This system has been described in detail previously [17]. In this study, the microendoscopy system can obtain images through direct contact between the rigid probe (Opto-Medic, Guangdong, China) and the cervix. The diameters of the circular fields of view are 250, 500, and 750 μm, corresponding to resolutions of 1, 2, and 4 μm, respectively, with a frame rate of about 30 fps.

In order to observe the morphology of cervical epithelial cells, we applied 1% methylene blue solution to stain the cervix. Methylene blue is an important biological stain, which can enhance the detection of lesions by increasing the contrast between normal and diseased tissues at the mucosal level without damaging tissue function, has been widely used in the endoscopic diagnosis of gastrointestinal tumors [20]. In addition, methylene blue can also be used as a tracer in surgery, a contrast agent for sentinel lymph nodes in radical tumor resection, and a photosensitizer for photodynamic therapy et al. [21]. Methylene blue has a long history of clinically safe use, and numerous clinical trials have verified its safety as a transient dye in vivo [20, 22, 23].

### Study protocol

The research protocol of this study was formulated according to previous research [17]. According to the inclusion and exclusion criteria, women with abnormal cervical screening results were formally enrolled in the study after signing the informed consent. Both conventional colposcopy and microendoscopy were performed by senior gynecologists.

During the in vivo imaging process, routine colposcopy was first performed according to the 2017 ASCCP colposcopy standard procedure [24]. After fully exposing the cervix, the colposcopic image characteristics of cervical tissue were observed under white light, dilute acetic acid staining, and 5% Lugol’s iodine solution staining to obtain the colposcopy impression. The colposcopy diagnosis was made according to the characteristics of colposcopy images.

Subsequently, high-resolution microendoscopic imaging was performed in the absence of staining. First, put the microendoscope probe gently close to the cervix, then moved the probe to observe the condition of various parts of the cervix, and finally put the probe deep into the cervical canal for observation. After imaging without staining, 1% methylene blue was applied to the cervix, stained for 1 min, and the above microendoscopic imaging process was performed again. The staining situation of the cervix and cervical canal was observed by moving the probe, focusing on the morphological characteristics of cells in abnormal parts. For the obtained microendoscopic images, the clock position of the lesion site and the microendoscopic diagnosis were recorded. Both conventional colposcopy diagnosis and microendoscopy diagnoses were recorded using the 2017 ASCCP colposcopy standard terminology for colposcopic practice, which was categorized as normal/benign, low grade, high grade, and cancer [24].

Finally, multi-point biopsies of abnormal cervical sites and cervical canal curettage were performed according to the 2017 ASCCP colposcopy guidelines [25]. In order to make the microendoscopy findings comparable with pathological biopsy specimens, we defined the dorsal side of the inserted microendoscope as the 6 o’clock orientation and the ventral side as the 12 o’clock orientation in the lithotomy position. The pathological diagnosis was made by experienced pathologists. All pathologists were blind to conventional colposcopy and microendoscopy images and the diagnosis of other pathologists.

## Results

### Patient characteristics

A total of 41 patients underwent routine colposcopy and microendoscopy during the study period. All patients obtained clear microendoscopic images and underwent cervical biopsy for the final pathological diagnosis. We used the histopathological diagnosis as the gold standard for evaluating the diagnostic features and accuracy of microendoscopy images. The clinical characteristics of the patients were shown in Table 1. The median age of enrolled patients was 43 years (range: 23 to 76 years), including 1 case of cervicitis, 5 cases of low-grade squamous intraepithelial lesion (LSIL), 31 cases of high-grade squamous intraepithelial lesion (HSIL), 1 case of adenocarcinoma in situ (AIS), 2 cases of squamous cell carcinoma (SCC), and 1 case of adenocarcinoma. No clinical adverse events occurred during the study.

**Table 1:** Patient characteristics

Patient characteristics	N = 41
Age (years)	
18–39	19
40–59	15
60 and older	7
median (range)	43 (23–76)
HPV	
High risk-HPV negative	1
HPV16 positive	22
HPV18 positive	3
Other high risk-HPV positive	15
Cytology	
Normal	9
ASC-US	10
ASC-H	4
LSIL	7
HSIL	8
SCC	2
Adenocarcinoma	1
Pathological diagnosis	
cervicitis	1
LSIL	5
HSIL	31
AIS	1
SCC	2
Adenocarcinoma	1

Abbreviations:

HPV: Human Papillomavirus, ASC-US: Atypical Squamous Cells-Undetermined Significance, ASC-H: Atypical Squamous Cells-cannot exclude High-grade squamous intraepithelial lesion, LSIL: Low-grade Squamous Intraepithelial Lesion, HSIL: High-grade Squamous Intraepithelial Lesion, SCC: Squamous Cell Carcinoma, AIS: Adenocarcinoma in Situ

### Diagnosis of cervical lesions under microendoscopy

We compared the consistency of microendoscopy diagnosis, conventional colposcopy diagnosis with pathological diagnosis separately. The data showed that the overall consistency of microendoscopy diagnosis with pathological diagnosis was 95.12% (39/41) and the overall consistency of conventional colposcopy diagnosis with pathological diagnosis was 65.85% (27/41). Furthermore, the contingency tables were used to show the specific diagnosis of different degree cervical lesions under microendoscopy and conventional colposcopy, which indicated that microendoscopy had advantages in the diagnosis of HSIL and more severe lesions (Supplementary Table S1-3).

### Cell morphological features

Microendoscopy can be close to the cervical surface for real-time imaging with cell resolution. Methylene blue staining can make the lesion area more obvious. Based on these technologies, we can intuitively observe the cell morphological features of different cervical lesions.

**Fig. 1:**
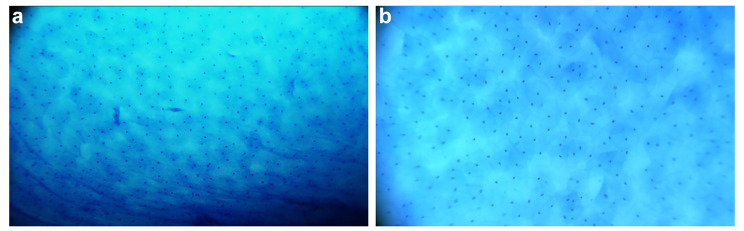
Representative methylene blue-stained microendoscopic images of Cervicitis (a) and LSIL (b)

The morphological abnormality of nuclei is the basic diagnostic feature to distinguish benign and malignant cells. We found that with the increase in the severity of cervical lesions, morphological abnormalities of the cervical nuclei became more and more obvious. In the normal cervix and cervicitis, methylene blue staining showed regularly arranged, round-like cervical epithelial cells with light blue cytoplasm and dark blue nuclei that appear as small, uniform circles (Fig. 1a). In LSIL patients, in addition to uniformly distributed round nuclei, a few enlarged or irregular nuclei were observed at the lesion site (Fig. 1b).

**Fig. 2:**
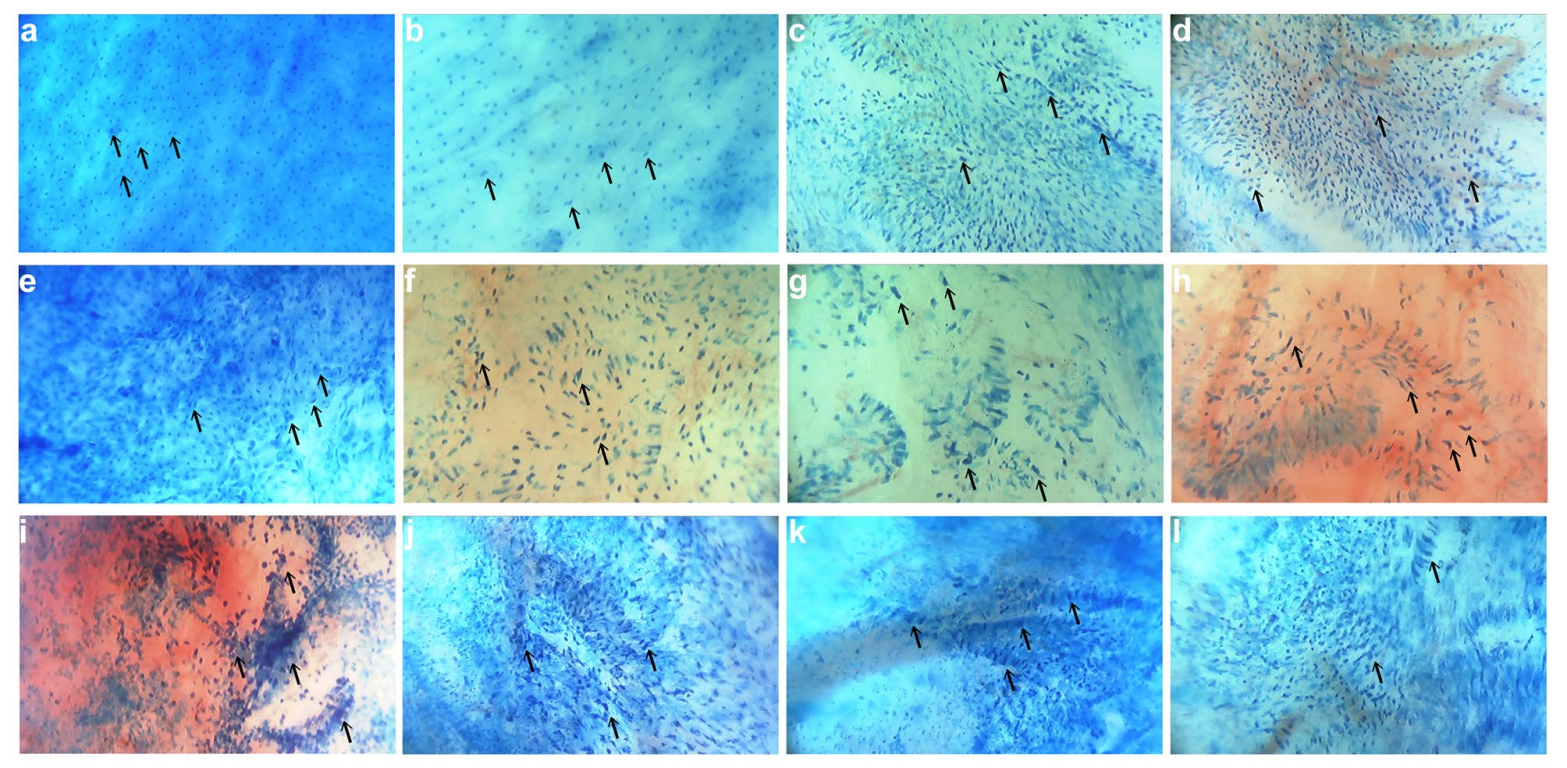
Representative methylene blue-stained microendoscopic images of HSIL, AIS, and SCC. (**a-d**) The abnormal cervical epithelial cells in HSIL were aggregated arranged with enlarged nuclei, irregular nuclear contours, and dark staining. (**e**) In AIS, crowded glandular epithelial cells with lightly stained lace-like cytoplasm and hyperchromatic enlarged pleomorphic nuclei were observed. (**f-l**) In SCC, cervical epithelial cells were disorganized, enlarged overlapping nuclei were irregular, with folds and dents, and abundant necrotic cell debris in the background

HSIL cells were often aggregated, with less and inconspicuous cytoplasm staining, and mainly characterized by nuclear pleomorphism. Specifically, most cells had enlarged nuclei, increased nuclear-to-cytoplasmic ratio, darkly stained nuclei, and irregular nuclear outlines (Fig. 2a-d). However, viewed as a whole, the HSIL nuclei were arranged in relatively regular directions. Compared with HSIL, AIS is not common. A representative microendoscopic image of AIS was shown in Fig. 2e. Crowded glandular epithelial cells can be seen, in which the cytoplasm was lightly stained and lace-like [26], and the enlarged nuclei were deeply stained with different shapes. In SCC patients, disordered cervical epithelial cells were observed, with a large number of necrotic cell debris in the background. The nuclei of cancer cells were significantly enlarged, the nuclear contour was highly irregular with folds and dents, and the intensity of nuclear staining was uneven, with a characteristic nuclear overlap phenomenon (Fig. 2f-l).

LSIL is generally used as a diagnostic category to describe cervical changes associated with transient HPV infection, while HSIL often represents a true precancerous lesion [27]. Some studies have pointed out that 30% of HSIL may progress to invasive cancer within 30 years [28]. Therefore, timely and accurate identification of HSIL and more serious lesions in the screening process is particularly important. However, biopsy under conventional colposcopy is invasive and can only be performed in superficial parts, which increases the chance of missed diagnosis [7]. In contrast, the probe of the microendoscope can be attached to the surface of the cervix and extended into the cervical canal for imaging, which makes up for the shortcomings of conventional colposcopy.

**Fig. 3:**
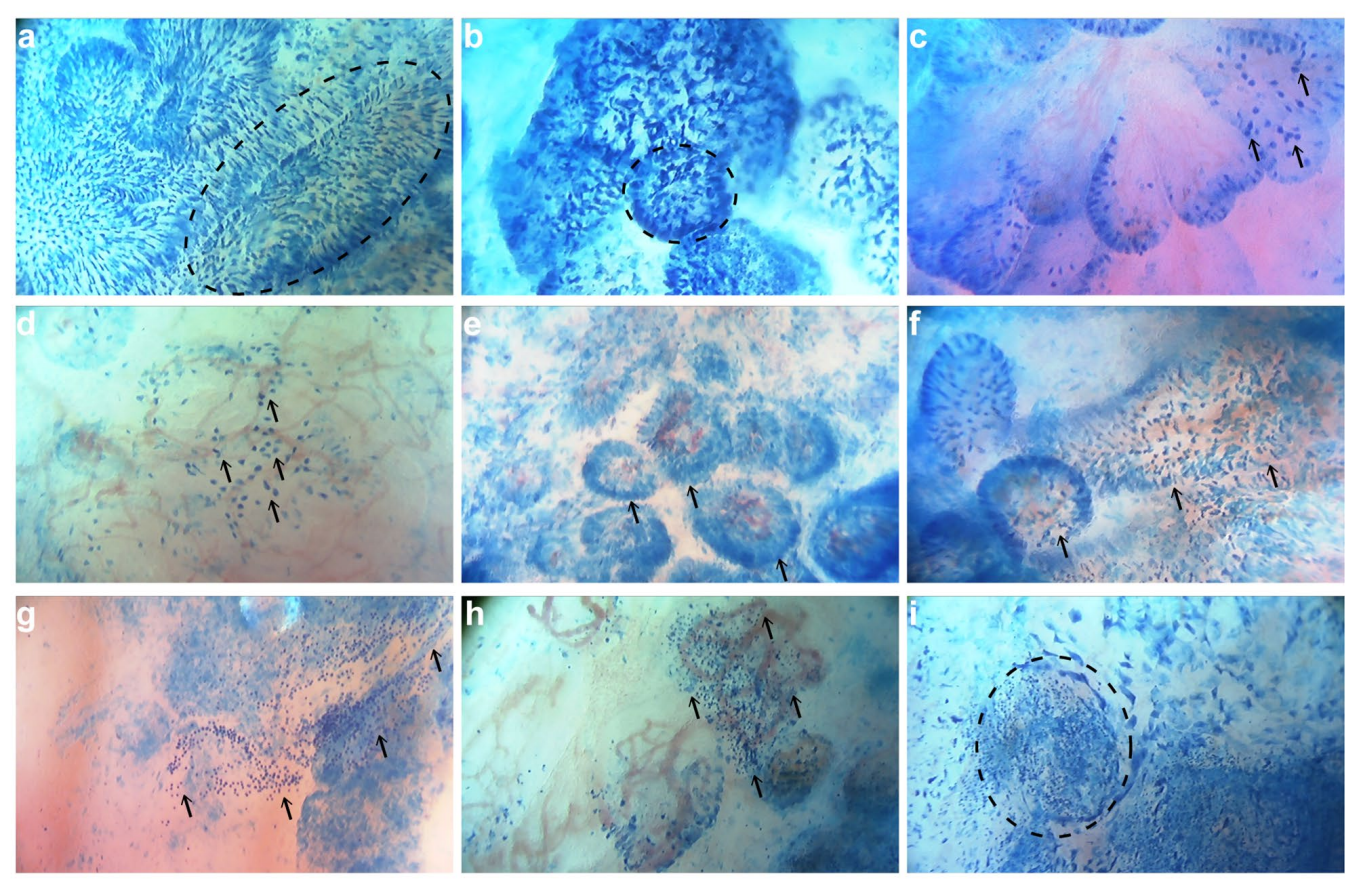
Characteristics of HSIL and more severe lesions after methylene blue staining under microendoscopy. (**a**) The columnar epithelial cells of the cervical canal were distributed as feathery or papillary aggregates in HSIL patients. (**b**) The columnar cells in the cervical canal were disordered, and the structure of cervical glands was obscure in the AIS patient. (**c**) The columnar epithelial cells were replaced by neoplastic squamous epithelial cells and the normal structure of cervical glands disappeared in SCC patients. (**d-f**) Enlarged and irregular hyperchromatic nuclei were seen on the surface of inverse mosaic vessels (**d**), cotton-like punctate blood vessels (**e**), and spiral-shaped atypical vessels (**f**) in HSIL, AIS, and SCC patients separately. (**g-h**) There was a large number of lymphocytes around the lesions of HSIL (**g**) and SCC (**h**). (**i**) Possible lymph node structures around cancer cells were observed in SCC patients

On this basis, we further analyzed the image features of HSIL and more severe lesions under microendoscopy. In HSIL patients, the columnar epithelial cells on the surface of the cervical canal were distributed in feathery or papillary aggregates, with indistinct cytoplasm and predominance of enlarged, elongated, strongly stained nuclei (Fig. 3a). While in the AIS patient, we observed a disordered arrangement of columnar cells in the endocervical canal, with crowded, enlarged heteromorphic nuclei, and the structure of the cervical glands was obscure (Fig. 3b). When the lesion progressed to invasive carcinoma, the normal structure of the cervical glands was lost, the cancer cells were scattered irregularly, and the enlarged cancer cell nuclei with different staining degrees had different morphology (Fig. 3c).

Malignant development of benign tumors is often associated with an angiogenic transition: the transition from a quiescent vasculature to a proliferative vasculature, so vascular heterogeneity is a hallmark of tumors [29]. In the previous study, we directly observed the characteristic pathological blood vessels in cervical precancerous lesions and cervical cancer through microendoscopy and used microvascular features as the bases for microendoscopic diagnosis [17]. In HSIL and more severe cervical lesions, morphologically abnormal cervical epithelial cells were frequently accompanied by pathological blood vessels. Enlarged and irregular hyperchromatic nuclei were seen on the surface of inverse mosaic vessels (Fig. 3d), cotton-like punctate blood vessels (Fig. 3e), and spiral-shaped atypical vessels (Fig. 3f).

In addition, we also observed many lymphocytes around the lesion site in HSIL and SCC (Fig. 3 g-h) and found possible lymph node structures around cancer cells (Fig. 3i). Such types of tumor-infiltrating lymphocytes accumulate in the tumor microenvironment and play an important role in the occurrence and development of cancer [30, 31].

Table 2 summarized the cell morphological features of different cervical lesions after methylene blue staining under microendoscopy.

**Table 2:** Cell morphological features of different cervical lesions after methylene blue staining under microendoscopy

Category	Cell morphological features
cervicitis	No abnormal features were found. Regularly arranged round-like cervical epithelial cells with light blue cytoplasm and dark blue nuclei that appear as uniform, small round.
LSIL	A few enlarged or irregular nuclei were present against a background of uniformly distributed round nuclei.
HSIL	Most cells had enlarged nuclei, increased nuclear-cytoplasmic ratio, darkly stained nuclei, and irregular nuclear contours, with relatively regular arrangement. Cervical canal columnar epithelial cells were distributed in feathery or papillary aggregates. Morphologically abnormal epithelial cells were often accompanied by pathological blood vessels. There were lymphocytes infiltration around the lesion.
AIS	Crowded glandular epithelial cells with lightly stained lacy-like cytoplasm and darkly stained enlarged nuclei. Disordered cervical canal columnar epithelial cells with crowded, enlarged heteromorphic nuclei, and the structure of the cervical glands was obscure. Morphologically abnormal epithelial cells were often accompanied by pathological blood vessels.
SCC	Disorganized cervical epithelial cells with numerous necrotic cell debris. Significantly enlarged nuclei, highly irregular nuclear contour with folds and dents, uneven intensity staining nuclei, and characteristic nuclear overlap. The normal structure of the cervical glands was lost, and cancer columnar epithelial cells were scattered irregularly with enlarged heteromorphic nuclei. Morphologically abnormal epithelial cells were often accompanied by pathological blood vessels. Possible lymph node structures surrounding the cancer cells.

Abbreviations:

LSIL: Low-grade Squamous Intraepithelial Lesion, HSIL: High-grade Squamous Intraepithelial Lesion, AIS: Adenocarcinoma in Situ, SCC: Squamous Cell Carcinoma

### Microendoscope identifies cellular features displayed in histological sections

**Fig. 4:**
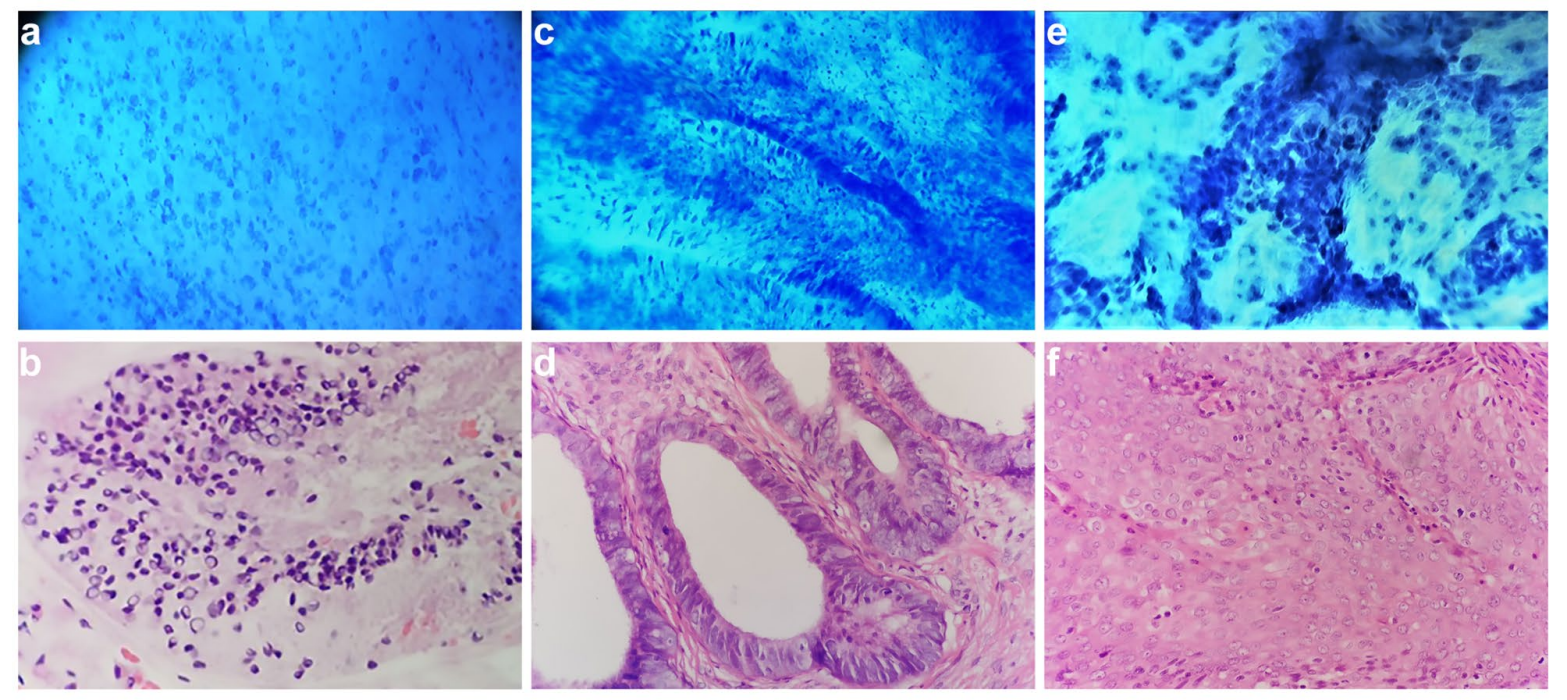
Microendoscopic pictures and H&E stained sections of HSIL and early cervical cancer patients were compared. Microendoscopy can reveal cellular features displayed in histological sections. (**a-b**) Abundant supramucoid cells were observed. The cytoplasm was full of mucus, and the nuclei were deeply stained, which were biased to the inner side of the cytoplasm under the microendoscopy (**a**) and tissue section (**b**) in the cervical canal of HSIL patients. (**c-d**) Abnormal columnar epithelial cells in a patient with adenocarcinoma in situ were observed in the cervical canal, with enlarged nuclei, intense staining, and neatly arranged feathers under microendoscopy (**c**) and H&E stained tissue pathological sections (**d**). (**e-f**) Squamous cell carcinoma of early cervical cancer patients showed heterogeneous features such as nuclear enlargement, hyperchromasia, and mitotic figures under microendoscopy (**e**) and H&E stained tissue pathological sections (**f**). All histopathology images were taken at the same magnification of × 400

Figure 4 showed microendoscopic images and corresponding H&E histopathological section images of patients with cervical tumors, which include HSIL, AIS, and SCC. In HSIL, the secretory cells on the surface of the cervical glands are functionally active and can secrete a large amount of mucus, which is one of the reasons why patients diagnosed with HSIL often have symptoms of increased vaginal secretions. In this case, a large number of relatively regularly arranged secretory cells with transparent cytoplasm and hyperchromatic nuclei can be observed under the microendoscopy (Fig. 4a). At the same time, a massive accumulation phenomenon of secretory cells was also observed in tissue sections from the same patient (Fig. 4b). The neoplastic columnar epithelial cells of glands within the cervical canal were observed in microendoscopic images of an AIS patient, with enlarged nuclei and strongly staining, and the glandular structure remained relatively intact (Fig. 4c). These microendoscopic appearances matched the corresponding H&E histopathological images (Fig. 4d). In SCC patients, microendoscopy can identify clusters of strongly stained cancer cells that infiltrate into surrounding areas and exhibit a heterogeneous feature (Fig. 4e). This microscopic feature was consistent with histopathology (Fig. 4f).

## Discussion

The anatomical location of the cervix is easier to expose than other organs. Therefore, the application of microendoscopy technology is more convenient, easier to be accepted by patients, and easier to promote, which meets the needs of large-scale cervical screening and diagnosis.

In this study, we applied the methylene blue cell staining technique in a microendoscopy system to achieve in vivo real-time imaging of cervical lesions for the first time, which is a great improvement over previous studies. We analyzed and summarized the cell morphological characteristics of methylene blue-stained cervical benign and neoplastic lesions in a cohort of 41 patients who underwent both conventional colposcopy and microendoscopy. In addition, we verified that microendoscopic images of HSIL and more severe cervical lesions could identify diagnostic features shown in the corresponding pathological sections.

The development of microendoscopy technology has opened up a new way for the early diagnosis of tumors. Some studies on the application of high-resolution microendoscopy in cervical cancer screening have been reported in the past. Chao Zhou et al. preliminarily investigated the image characteristics of different cervical lesions by three dimensional imaging of ex vivo cervical samples using ultrahigh-resolution optical coherence microscopy, but the in vivo application of this technology has not yet been achieved [7]. Rebecca R. Richards-Kortum et al. developed a high-resolution fluorescence microendoscope system, which has a fiber-optic probe that images cervical epithelial cell nuclei, providing morphological information associated with high-grade dysplasia [16, 32, 33]. This system requires the application of proflavine to fluorescently stain cervical epithelial cell nuclei. However, proflavine, which can induce DNA mutations by inserting between nucleic acid base pairs, is a highly hazardous and mutagenic dye [34]. In the United States, the use of proflavine on humans is strictly limited [35]. This has limited the widespread clinical application of the fluorescence endoscopic microscope system.

Different from previous studies, the microendoscope we developed was an optical microscope, which relied on the high-intensity white light source and the high-resolution camera on the probe to image after being processed by the optical system, so it did not rely on proflavine as a local contrast agent for fluorescence staining of cervical epithelial cell nuclei. In this study, methylene blue solution was used to stain cervical epithelial cells for the first time. Methylene blue, a phenothiazine derivative dye, has a small molecular weight, can diffuse quickly into tissues, and has the ability to bind DNA and RNA, and is widely used as a nucleic acid dye [36]. Previous in vivo studies in animal models and clinical experiments have not only demonstrated the non-toxicity of methylene blue, but also demonstrated that methylene blue can improve tissue status in addition to its use as a dye [23, 37]. Methylene blue has been approved by the US Food and Drug Administration for clinical treatment, including the treatments of methemoglobinemia, an antiseptic in clinical therapy and the dye as a tissue tracer for surgery and endoscopy, etc. [38]. In addition, some clinical studies in recent years have also reported the effectiveness of methylene blue staining in the detection of precancerous lesions and cancers in the oral cavity and digestive tract [39, 40]. Therefore, the safety and feasibility of in vivo transient application of a low-dose methylene blue solution as a cell staining dye for microendoscopy of the cervix in this study were guaranteed.

In conventional colposcopy, abnormal colposcopic findings such as acetowhite, vascular patterns, margins or border, Lugol staining, etc. which often appear in the cervical transformation zone, are mainly used to diagnose high-grade lesions and invasive carcinomas [24]. However, our previous clinical practice found that there were some cases that did not respond significantly to acetic acid or Lugol’s iodine staining during colposcopy, and it was difficult to recognize these diagnostic features in patients where the squamocolumnar junction was not fully visible [17]. In this study, we demonstrated the feasibility of using methylene blue staining under microendoscopy to triage HSIL and more severe lesions. In cervical benign lesions and LSIL, abnormal cells were rare under microendoscopy. When the lesion progressed to HSIL, we observed characteristic changes in microendoscopic images of the cervix: Neoplastic cervical epithelial cells with enlarged, pleomorphic, hyperchromatic nuclei gradually infiltrated the surrounding area tissues with the increase in the severity of the lesion; columnar epithelial cells on the surface of cervical glands in the cervical canal exhibited characteristic feathery or papillary arrangement, accompanied by elongated and hyperchromatic nuclei; Columnar epithelial cells were replaced by neoplastic squamous epithelial cells and the normal structure of cervical glands disappeared, when the lesions involved the glands; tumor cells clustered around pathological blood vessels under microendoscope, suggesting the characteristic vascular heterogeneity of the tumor; moreover, there was lymphocyte infiltration around cervical neoplastic cells. The host immune response plays a key role in determining whether HPV exposure leads to infection, persistence, progression, and eventual carcinogenesis, and this characteristic accumulation of tumor-infiltrating lymphocytes within the cervical tumor microenvironment suggests that aggressive cancers are highly immunogenic. Infiltrating CD3 + and CD4 + T-cells have a positive effect on cervical cancer prognosis, whereas regulatory T-cells infiltration increases with cervical lesion progression [41]. In addition, for the squamocolumnar junction, which was not fully visible in conventional colposcopy, the probe of the microendoscope could enter the cervical canal for imaging, which effectively avoided missed diagnosis.

However, this study still has some limitations, which need to be further explored. Firstly, as a preliminary exploratory report, the sample size included in this study was relatively small. Although we have described the cell morphological features of cervicitis, LSIL, HSIL, AIS, and SCC after methylene blue staining under microendoscope, respectively, the number of each lesion cases was relatively small, especially AIS and SCC, so further clinical trials with a large number of samples are needed to establish, improve, and evaluate the diagnostic criteria for cervical lesions by methylene blue cell staining under microendoscope. Secondly, the current microendoscopy system solved the problem of missed diagnosis and excessive biopsy of conventional colposcopy, but it still required professional gynecologists to make a visual diagnosis based on image features. In the follow-up study, we will attempt to integrate artificial intelligence technology with the current microendoscopy system to expand the functions and applications of microendoscopy and develop machine learning algorithms to realize the intelligent analysis of microendoscopic images to assist clinical decision-making. Finally, in future research, the real-time quantitative analysis of the lesion-related features in microendoscopic cervical cell images, such as nuclear-cytoplasmic ratio, abnormal nuclear density (i.e., number of abnormal nuclei/mm2), and other parameters, can be performed by combining fully automatic image processing algorithms [42, 43]. This approach will improve the accuracy and reproducibility of diagnosis and provide an objective and quantitative standard for clinicians to diagnose cervical lesions using the microendoscopy system.

In conclusion, we reported the first study of in vivo real-time imaging of cervical lesions using a combination of a high-resolution microendoscopy system and methylene blue cell staining technique. By analyzing the methylene blue-stained microendoscopic images from 41 patients, we determined the diagnostic cell morphological features of different cervical lesions, including cervicitis, LSIL, HSIL, AIS, and SCC. Our study demonstrated that the microendoscopic methylene blue cell staining technique could visualize microscopic cellular diagnostic features consistent with histopathology in HSIL and more severe lesions. The microendoscopic methylene blue cell staining technique deserves future large-scale clinical studies to validate diagnostic performance. This study preliminarily practiced the application of the microendoscopy imaging system combined with the methylene blue cell staining technology in cervical precancerous lesions and cervical cancer screening and provided the basis for a novel clinical strategy of in vivo non-invasive optical diagnosis technology to triage women with abnormal cervical screening results.

## Electronic supplementary material

Below is the link to the electronic supplementary material.

Supplementary Material 1

## Data Availability

All data generated or analysed during this study are included in this published article [and its supplementary information files].

## List of abbreviations

AIS: Adenocarcinoma in situ
ASCCP: American society for colposcopy and cervical pathology
CIN: Cervical intraepithelial neoplasia
HPV: Human papillomavirus
HSIL: High-grade squamous intraepithelial lesion
LSIL: Low-grade squamous intraepithelial lesion
SCC: Squamous cell carcinoma

## References

[1] Sung H, Ferlay J, Siegel RL (2021). Global Cancer Statistics 2020: GLOBOCAN estimates of incidence and Mortality Worldwide for 36 cancers in 185 countries. CA Cancer J Clin.

[2] Grigsby PW, Massad LS, Mutch DG (2020). FIGO 2018 staging criteria for cervical cancer: impact on stage migration and survival. Gynecol Oncol.

[3] Wenzel HHB, Bekkers RLM, Lemmens VEPP (2021). No improvement in survival of older women with cervical cancer-A nationwide study. Eur J Cancer.

[4] Jeronimo J, Massad LS, Castle PE (2007). Interobserver agreement in the evaluation of digitized cervical images. Obstet Gynecol.

[5] Stoler MH, Vichnin MD, Ferenczy A (2011). The accuracy of colposcopic biopsy: analyses from the placebo arm of the Gardasil clinical trials. Int J Cancer.

[6] Fachetti-Machado G, Figueiredo-Alves RR, Moreira MAR (2022). Performance of three colposcopic images for the identification of squamous and glandular cervical precursor neoplasias. Arch Gynecol Obstet.

[7] Zeng X, Zhang X, Li C (2018). Ultrahigh-resolution optical coherence microscopy accurately classifies precancerous and cancerous human cervix free of labeling. Theranostics.

[8] Hunt B, Fregnani JHTG, Schwarz RA (2018). Diagnosing cervical neoplasia in rural Brazil using a Mobile Van equipped with in vivo Microscopy: a Cluster-Randomized Community Trial. Cancer Prev Res.

[9] Stanley MA, Sterling JC (2014). Host responses to infection with human papillomavirus. Curr Probl Dermatol.

[10] Schiffman M, Doorbar J, Wentzensen N (2016). Carcinogenic human papillomavirus infection. Nat Rev Dis Primers.

[11] Della Fera AN, Warburton A, Coursey TL (2021). Persistent Hum Papillomavirus Infect Viruses.

[12] Koliopoulos G, Nyaga VN, Santesso N (2017). Cytology versus HPV testing for cervical cancer screening in the general population. Cochrane Database Syst Rev.

[13] Lopez DR, Sgroi D, Krishnamourthy S (2022). Is Real-Time Microscopy on the Horizon? A brief review of the potential future directions in clinical breast Tumor Microscopy implementation. Virchows Arch.

[14] Louie JS, Richards-Kortum R, Anandasabapathy S (2014). Applications and advancements in the use of high-resolution Microendoscopy for detection of gastrointestinal neoplasia. Clin Gastroenterol H.

[15] Wu CZ, Gleysteen J, Teraphongphom NT (2018). In-vivo optical imaging in head and neck oncology: basic principles, clinical applications and future directions. Int J Oral Sci.

[16] Hunt B, Fregnani JHTG, Brenes D (2021). Cervical lesion assessment using real-time microendoscopy image analysis in Brazil: the CLARA study. Int J Cancer.

[17] Guo J, Fu L, Zhao J (2020). The value of microendoscopy in the diagnosis of cervical precancerous lesions and cervical microinvasive carcinoma. Arch Gynecol Obstet.

[18] Alvarado-Kristensson M, Rossello CA (2019). The Biology of the Nuclear envelope and its implications in Cancer Biology. Int J Mol Sci.

[19] Fischer EG (2020). Nuclear morphology and the Biology of Cancer cells. Acta Cytol.

[20] Di Stefano AFD, Radicioni MM, Vaccani A (2018). Methylene blue MMX (R) tablets for chromoendoscopy. Bioavailability, colon staining and safety in healthy volunteers undergoing a full colonoscopy. Contemp Clin Trials.

[21] Lim DJ (2021). Methylene Blue-Based Nano and Microparticles: fabrication and applications in photodynamic therapy. Polym (Basel).

[22] Tariq B, Simon SR, Pilz W (2021). Evaluating the safety of oral methylene blue during swallowing assessment: a systematic review. Eur Arch Otorhinolaryngol.

[23] Repici A, Ciscato C, Wallace M (2018). Evaluation of genotoxicity related to oral methylene blue chromoendoscopy. Endoscopy.

[24] Khan MJ, Werner CL, Darragh TM (2017). ASCCP Colposcopy Standards: role of Colposcopy, benefits, potential Harms, and terminology for colposcopic practice. J Low Genit Tract Dis.

[25] Wentzensen N, Schiffman M, Silver MI (2017). ASCCP Colposcopy Standards: risk-based Colposcopy Practice. J Low Genit Tract Dis.

[26] Alrajjal A, Pansare V, Choudhury MSR (2021). Squamous intraepithelial lesions (SIL: LSIL, HSIL, ASCUS, ASC-H, LSIL-H) of Uterine Cervix and Bethesda System. Cytojournal.

[27] Darragh TM, Colgan TJ, Thomas Cox J (2013). The Lower Anogenital squamous terminology standardization project for HPV-associated lesions: background and consensus recommendations from the College of American Pathologists and the American Society for Colposcopy and Cervical Pathology. Int J Gynecol Pathol.

[28] Egemen D, Cheung LC, Chen X (2020). Risk estimates supporting the 2019 ASCCP risk-based Management Consensus Guidelines. J Low Genit Tract Dis.

[29] De Palma M, Biziato D, Petrova TV (2017). Microenvironmental regulation of tumour angiogenesis. Nat Rev Cancer.

[30] Fridman WH, Galon J, Pages F (2011). Prognostic and predictive impact of intra- and Peritumoral Immune infiltrates. Cancer Res.

[31] Schreiber RD, Old LJ, Smyth MJ (2011). Cancer Immunoediting: integrating immunity’s Roles in Cancer suppression and Promotion. Science.

[32] Parra SG, Rodriguez AM, Cherry KD (2019). Low-cost, high-resolution imaging for detecting cervical precancer in medically-underserved areas of Texas. Gynecol Oncol.

[33] Parra SG, Lopez-Orellana LM, Molina Duque AR (2020). Cervical cancer prevention in El Salvador: a prospective evaluation of screening and triage strategies incorporating high-resolution microendoscopy to detect cervical precancer. Int J Cancer.

[34] Gatasheh MK, Kannan S, Hemalatha K (2017). Proflavine an acridine DNA intercalating agent and strong antimicrobial possessing potential properties of carcinogen. Karbala Int J Mod Sci.

[35] Nedu ME, Tertis M, Cristea C (2020). Comparative study regarding the Properties of Methylene Blue and Proflavine and their optimal concentrations for in Vitro and in vivo applications. Diagnostics.

[36] Hossain M, Kumar GS (2009). DNA intercalation of methylene blue and quinacrine: new insights into base and sequence specificity from structural and thermodynamic studies with polynucleotides. Mol Biosyst.

[37] Taghavi SA, Membari ME, Eshraghian A (2009). Comparison of chromoendoscopy and conventional endoscopy in the detection of premalignant gastric lesions. Can J Gastroenterol.

[38] Ginimuge PR, Jyothi SD (2010). Methylene blue: revisited. J Anaesthesiol Clin Pharmacol.

[39] Riaz A, Shreedhar B, Kamboj M (2013). Methylene blue as an early diagnostic marker for oral precancer and cancer. Springerplus.

[40] Hirose T, Kakushima N, Furukawa K (2021). Endocytoscopy is useful for the diagnosis of superficial nonampullary duodenal epithelial tumors. Digestion.

[41] Litwin TR, Irvin SR, Chornock RL (2021). Infiltrating T-cell markers in cervical carcinogenesis: a systematic review and meta-analysis. Br J Cancer.

[42] Shin D, Protano MA, Polydorides AD (2015). Quantitative analysis of high-resolution microendoscopic images for diagnosis of esophageal squamous cell carcinoma. Clin Gastroenterol Hepatol.

[43] Tan MMC, Bhushan S, Quang T (2021). Automated software-assisted diagnosis of esophageal squamous cell neoplasia using high-resolution microendoscopy. Gastrointest Endosc.

