# Supplementary material for: Microendoscopy in vivo for the pathological diagnosis of cervical precancerous lesions and early cervical cancer
Source: Infect Agent Cancer. 2023 Apr 26;18:26. doi: 10.1186/s13027-023-00498-8 (PMC10134531; doi:10.1186/s13027-023-00498-8)
Supplement: Supplementary file 1 — Supplementary Material 1 [file 13027_2023_498_MOESM1_ESM.docx]

Supplementary Table S1 Correlation between microendoscopy diagnosis with histological diagnosis.

| Microendoscopy | Pathological biopsy | | |
| --- | --- | --- | --- |
|  | Cervicitis | LSIL | ≥HSIL |
| Cervicitis | 1 | 0 | 0 |
| LSIL | 0 | 4 | 0 |
| ≥HSIL | 0 | 1 | 35 |

Supplementary Table S2 Correlation between colposcopy diagnosis with histological diagnosis.

| Colposcopy | Pathological biopsy | | |
| --- | --- | --- | --- |
|  | Cervicitis | LSIL | ≥HSIL |
| Cervicitis | 1 | 0 | 3 |
| LSIL | 0 | 3 | 7 |
| ≥HSIL | 0 | 2 | 25 |

Supplementary Table S3 Correlation between colposcopy diagnosis with microendoscopy diagnosis.

| Colposcopy | Microendoscopy | | |
| --- | --- | --- | --- |
|  | Cervicitis | LSIL | ≥HSIL |
| Cervicitis | 1 | 0 | 3 |
| LSIL | 0 | 3 | 7 |
| ≥HSIL | 0 | 1 | 26 |
